# Supplementary material for: Are carbon emissions trading and green financial instruments synergistic? -Comprehensive quantitative research based on content analysis
Source: PLoS One. 2024 Mar 7;19(3):e0298601. doi: 10.1371/journal.pone.0298601 (PMC10919601; doi:10.1371/journal.pone.0298601)
Supplement: S1 Data — (ZIP) [file pone.0298601.s001.zip › Data/地级市Z_F工作报告.zip!/─┌├╔╣┼/╬┌└╝▓∞▓╝╩╨/└┤╫╘╣1⁄2╓┌║┼í╛┬φ┐╦╩2╛▌═°í┐_2015.docx]

各位代表：

现在，我代表市人民政府向大会报告工作，请予审议，并请各位政协委员和列席会议的同志们提出意见。

过去一年，是乌兰察布市发展进程中充满挑战，又富有成效的一年。在自治区党委、政府和市委的坚强领导下，在乌兰察布市人大和政协的监督支持下，市政府认真贯彻习近平总书记系列重要讲话精神，坚持以“8337”发展思路为统领，团结带领全市各族人民，统筹推进稳增长、调结构、促改革、惠民生，经济社会各项事业取得新业绩。

（一）综合实力明显增强。面对宏观经济持续下行的严峻考验，全市上下坚定信心、主动作为，加强调度分析，及时解决问题，经济发展呈现出稳中有进、稳中提质的良好态势。预计地区生产总值完成898亿元，增长8.5%，公共财政预算收入完成51亿元，增长20%，增速位居全区前列。重点工程、重大项目稳步推进，实施亿元以上项目349个，10亿元以上项目56个。预计全社会固定资产投资完成944亿元，增长18%，社会消费品零售总额完成265亿元，增长11%。

（二）改革开放取得突破。以经济体制改革为牵引，各领域、各方面135项改革全面推开。简政放权力度进一步加大。文化工商质监食药管理体制和机构改革积极推进。扩权强县试点工作全面启动。市本级取消和下放行政审批事项99项，精简率48%，行政事业性收费取消111项、停征34项。审批和服务类项目即时办结率分别达到30%和90%。推进工商注册制度便利化，各类市场主体和注册资本分别增长16%和31%，创历史新高。农村牧区改革深入推进。土地、草牧场承包经营权确权登记颁证试点工作启动，土地、草牧场流转面积分别达到303万亩、384万亩。财税金融领域改革成效明显。政府和部门预算、“三公经费”预决算全部向社会公开。“营改增”试点范围进一步扩大。农信社改革深入推进，乌兰察布农商银行、察右前旗农商银行挂牌运营。区域合作取得积极进展。蒙晋冀（乌大张）长城金三角合作区建设正式启动，京蒙对口帮扶深入推进，集宁海关正式开关，开创了改革开放新局面。

（三）产业结构不断优化。坚持稳增长、促转型相结合，把转变发展方式放在更加重要位置，产业结构调整取得新进展。现代农牧业加快发展。着力完善产业体系、服务体系、农畜产品质量安全体系，设施农业和规模养殖业效益不断提升。马铃薯种薯达到100万亩，建成250公里马铃薯产业带，规模化种植比例达到1/3。冷凉蔬菜达到77万亩。建成“百千万”养殖园区156处，牧业年度牲畜总头数802万头（只）。引进雏鹰、田牧等一批产业化重点项目，农畜产品加工转化率由48%提高到50%。新增国家农业综合标准化示范项目2个，国家级示范合作社达到19个。认证无公害、绿色产品25个。“丰镇月饼”、“卓资熏鸡”通过国家地理标志证明商标初审核准。工业经济稳中有进。积极应对资源环境约束加剧、市场需求不足、企业效益下滑等困难，实施电力补贴、减免涉企收费等综合政策，为企业降低用电成本12.6亿元，工业用电量增长11%，停产半停产企业启动63%。坚持淘汰与升级并重，拆除1.25万千伏安以下矿热炉25台，大型环保矿热炉产能占电石铁合金总产能的75%。切实加强园区建设管理，当年完成基础设施投资20亿元，园区产值占规上工业总产值的90%，3个园区营业收入超百亿元。预计工业固定资产投资和规上工业增加值增长18%和11%，居全区前列。现代服务业态势良好。区域性商贸物流中心建设成效明显。

西北煤炭电子交易平台试运行，乌兰察布马铃薯交易中心筹备上线，第四届马铃薯展洽会成功举办，土牧尔台制革基地功能逐步完善，“三个交易中心”实现营业收入77亿元。建成283家便民连锁超市、23个配送中心，维多利、红星美凯龙等大型城市商业综合体投入运营，城乡市场体系更加完善。旅游观光休闲度假基地建设步伐加快。全市旅游产业规划体系基本形成，“一心两翼三境区”格局初步建立，市旅游集团公司组建运营。凉城县旅游业被列为自治区70周年大庆献礼工程，辉腾锡勒等重点景区整合初见成效。我市被中国气象学会命名为“中国草原避暑之都”。全年接待游客1000万人次，实现旅游收入90亿元，分别增长25%和23%。金融支持实体经济力度加大。市本级累计投入各类担保资金1.8亿元，撬动银行贷款16.5亿元，三农三牧、小微企业金融服务得到加强。积极创新“助保贷”、“薯业贷”等金融产品，金融机构存贷比达到70%，首次达到全国平均水平。

（四）城乡面貌显著变化。中心城区各项规划协同推进，建设用地实现控规全覆盖。村镇体系和村庄布局规划编制完成，乡镇总体规划实现全覆盖。规划实施工作进入自治区前列。城镇化率提高到46%。以提高综合承载能力为重点，宜居城镇建设取得实效。实施城建项目767项，完成投资152亿元。城市人均道路面积26.5平方米，集中供热普及率81.5%。中心城区人均公园绿地面积35平方米，建成区绿化覆盖率39%，万人公共交通拥有量9.7标台，均达到或超过自治区平均水平。“五城联创”进入实质性阶段，中心城区创建国家园林城市通过自治区初审。“三山两河”生态景观获得自治区“人居环境范例奖”，市中心医院门诊楼、刑侦指挥中心大楼分获国家建筑工程鲁班奖和国家优质工程。以“十个全覆盖”工程为重点，美丽村镇建设成效显著。投入资金20亿元，完成936个自然村的“十个全覆盖”任务，惠及30万农牧民，农村牧区面貌发生历史性变化。丰镇市隆盛庄被命名为中国历史文化名镇，四子王旗大庙等3个村列入国家传统村落名录，集宁区白海子等14个镇被评为国家级重点示范镇。以交通、电力、水利基础设施为重点，发展条件明显改善。乌兰察布汽车客运总站投入运营。集宁南绕城等一批重点公路建成通车，新增高速公路215公里，总里程位居全区首位。嘎查村通沥青水泥路率达到76%，通班车率达到97%。呼张客专完成投资26亿元。支线机场建设工程基本完工。隆胜水库等重点水利项目加快推进。解决13万人饮水安全问题。实施110千伏以上电网项目16个，新增变电容量121万千瓦。

（五）生态环境全面改善。坚持把生态环境保护与建设作为硬约束、硬指标，着力呵护碧水蓝天，建设美丽乌兰察布。实施国家重点生态工程93万亩、自治区重点区域绿化工程15万亩，完成草原生态补奖项目3600万亩。落实京津冀及周边大气污染防治行动计划，开展环境保护、安全生产隐患排查整治“百日行动”。APEC会议期间空气质量保障工作圆满完成。节能减排任务全面完成。

（六）民生保障更加有力。始终把改善民生作为政府工作的出发点和落脚点，优先保障重点民生支出。预计城乡居民收入分别增长10%和12%，均高于经济增速。“四个一”民生工程和“十件实事”全面完成。扶贫攻坚工程扎实推进。“三到村三到户”精准扶贫深入实施，完成144个重点贫困嘎查村整村推进任务。开启政府主导、市场运行的扶贫新模式，发放金融扶贫富民贷款10亿元，惠及贫困户1.4万户、龙头企业22家。实施生态移民扶贫搬迁1万多人。10万贫困人口稳定脱贫。就业创业工程成效显著。农村牧区劳动力转移就业36万人次，新增城镇就业1.7万人，城镇登记失业率控制在4%以内。驻呼市、包头外出务工人员服务中心，被评为全国人社系统诚信服务示范单位。“乌兰美”跻身全区家政服务十佳企业和自治区级劳务品牌，集宁国际皮革城成为自治区级创业示范园。百姓安居工程加快实施。开工建设各类保障性住房4.8万套，基本建成3.7万套。完成农村牧区危房改造3.9万户，建设互助幸福院2410户，均超额完成目标任务。保障性安居工程任务量居全区首位，农危改成为全区典范，互助养老政策被评为“中国十大创新社会福利政策”。社会保障水平不断提高。社会保险扩面任务全面完成。企业退休人员养老保险待遇稳步提高。城乡医疗保险水平明显提升，城镇职工和居民住院实际报销比例分别达到77%、67%，新农合市县乡三级住院实际报销比例分别达到63%、73%、91%。大病保障救助机制全面建立，惠及城乡居民近40万人次。城乡低保和五保供养水平进一步提高。在全区率先实行困境儿童救助政策。我市被民政部评为“五星级慈善城市”，跻身“中国城市公益慈善百强榜”。

（七）社会事业全面进步。教育事业快速发展。义务教育和职业教育经费增速全区第一。新建23所农村牧区学校校舍，北京八中分校建成使用，义务教育学校标准化水平达到60%以上。新建城镇幼儿园11所，学前三年入正规园率提高到70%。“三项改革”深入推进，集宁师院附中、凉城一中被评为自治区示范高中。卓资县职业教育集团挂牌成立。民族学校蒙语授课学生率先实现免费教育全覆盖。医疗卫生服务水平明显提高。公立医院改革进展顺利，凉城等5所县级医院被确定为国家和自治区级试点。医疗基础设施建设步伐加快，建成县乡村医疗卫生场所320个，市第四医院新院区投入使用。出生人口素质不断提高。食品药品实现“网格化”监管全覆盖，被自治区确定为首批创建国家级食品安全示范市。基本公共卫生服务和人口计生、食药监管工作跃居全区前列。科技文体事业全面发展。科技创新步伐明显加快，实施国家、自治区科技项目33项。冷凉蔬菜院士工作站引进推广一批新品种、新技术。瑞盛新能源公司被认定为自治区级天然石墨工程研究中心，与清华大学合作实施石墨烯等重大科研项目。公共文化服务体系进一步完善，市博物馆、图书馆投入使用，文化惠民工程深入实施。体育事业蓬勃发展，万人徒步行等33项全民健身活动成为自治区品牌赛事，自治区十四运筹备工作全面启动。社会管理不断创新。“平安乌兰察布”建设扎实推进，刑事案件下降30%，人民群众安全感和满意度明显提升。安全生产形势保持平稳。信访工作不断加强。“社区场所建设年”活动成效显著，建成标准化社区203个，集宁文化路等3个社区荣获“全国和谐社区建设示范单位”称号。民族宗教工作取得新成绩，察右后旗政府等2个集体被授予“全国民族团结进步模范集体”。圆满完成第三次全国经济普查。支持军队和国防后备力量建设，双拥创建成果得到巩固。统计、人防、地震、妇女儿童、残疾人等工作长足发展。

（八）政府自身建设不断加强。扎实开展党的群众路线教育实践活动，严格落实中央八项规定，认真查摆“四风”方面的突出问题，着力抓好整改落实和建章立制，进一步密切了党群干群关系，行政机关和干部作风明显好转。全面清理超标办公用房和公务用车，全市行政事业单位“三公经费”下降21%。自觉接受市人大及其常委会的法律监督、工作监督，主动接受政协民主监督，认真落实“十二五”规划中期实施情况等审议意见，人大代表建议和政协提案全部办结。政务公开、行政监察、审计监督和依法行政工作不断加强，勤政廉政建设成效明显。

各位代表，过去一年的成绩来之不易。这是自治区党委、政府和市委科学决策、正确领导的结果，是市人大、政协加强监督、大力支持的结果，更是全市各族干部群众锐意进取、奋力拼搏的结果。在此，我代表市人民政府，向全市各族干部群众，向人大代表、政协委员，各民主党派和人民团体，向所有支持乌兰察布建设发展的同志们、朋友们，表示衷心的感谢并致以崇高的敬意！

在肯定成绩的同时，我们也清醒地认识到，我市经济社会发展还存在一些困难和问题。一是经济下行压力仍然较大。宏观经济环境趋紧，市场主体活力不足，投资稳定增长难度加大，部分企业生产经营比较困难。二是结构性矛盾相对突出。产业结构单一，创新能力不足；基础设施承载能力不强，城乡区域发展不平衡。三是民生有待进一步改善。社会事业发展相对滞后，就业和社会保障压力较大，部分群众生活比较困难。四是政府自身建设还需不断加强。一些单位职能转变还不到位，部分干部依法行政和为民服务能力有待提高。对于这些问题，我们要认真加以解决。2015年是全面深化改革的关键之年，是全面推进依法治国的开局之年，也是全面完成“十二五”规划的收官之年，做好今年工作意义重大。我们要按照中央、自治区的分析判断和部署要求，坚定信心，主动作为，准确把握新常态的大背景、大趋势，积极抢抓新机遇，毫不动摇地抓好发展这个第一要务，努力在新常态下谋求新发展、实现新跨越、开创新局面。

今年政府工作的总体要求：全面贯彻党的十八大、十八届三中、四中全会和习近平总书记系列重要讲话精神，深入落实自治区党委九届十三次全委会和市委三届五次全委（扩大）会议精神，坚持稳中求进工作总基调，主动适应经济发展新常态，以“三篇文章”为统领，着力建设六个基地，培育四大新兴产业；以提高经济发展质量和效益为中心，突出创新驱动，狠抓改革攻坚，深化区域合作，加强民生保障，推动经济跨越发展、社会和谐稳定。

主要预期目标是：地区生产总值增长8%，公共财政预算收入增长8%，固定资产投资增长13%，社会消费品零售总额增长11%，城乡居民收入分别增长9%和10%。

重点抓好九个方面的工作。

（一）深化改革开放，增强发展活力和动力

继续深化重点领域和关键环节改革。围绕转变政府职能，进一步简政放权，建立权力清单、责任清单、负面清单，给市场让出更大空间。市县两级政务服务大厅全部实现网上审批，苏木乡镇综合便民服务中心实现全覆盖。审批和服务项目即时办结率分别提高到50%以上和100%。加快旗县公共资源交易市场和中小企业公共服务平台建设。开展政府购买公共服务试点。围绕完善现代市场体系，深化“先照后证”工商登记制度改革，推行“同城异地（所）”办照，实现“三证合一”。开展企业信用信息公示工作，大型商场推行诚信管理模式，打造全区首个诚信建设平台。深化金融体制改革，争取1至2家农信社转制为农商银行。创新“助保贷”、保险保证贷款、农村牧区“三权”抵押贷款等金融产品。围绕加强融资能力建设，推进政府投融资平台转型发展，推行PPP合作模式，引入社会资本投资公共服务领域。用好信贷政策，进一步扩大保障性住房、基础设施建设等项目的融资规模。围绕加快农牧业现代化进程，完成草原确权承包和基本草原划定工作，全面开展土地承包经营权确权登记颁证工作。围绕加强财税管理，认真执行新《预算法》，推进预算管理制度改革。建立政府全口径预算，强化预算约束，政府和部门预决算向社会全面公开。加强政府项目投资评审，严格财政资金绩效管理，提升政府债务管控和化解水平。

做好“三篇文章”，全面提升对外开放水平。把“三篇文章”作为对接国家“一带一路”建设和京津冀协同发展的主战略，努力拓宽发展的新空间、新路径。加快推进蒙晋冀（乌大张）长城金三角建设。编制完成长城金三角合作区规划，力争进入国家区域合作战略体系。完善协调机制，推进基础设施、产业发展、公共服务、生态环境等领域合作。积极融入京津冀协同发展。依托环渤海市长联席会议和京蒙对口帮扶机制，探索园区共建、项目共管、收益共享的合作模式，发展“飞地经济”，建成京津产业转移的示范区、产业升级的助力区、产业发展的拓展区。不断扩大向北开放。争取将善丹呼日勒口岸列入国家“十三五”规划。加快海关监管场所建设。发展金属、煤炭、木材等进口资源加工产业。扩大劳务输出、境外投资、跨境旅游，开拓俄蒙欧市场。

（二）发挥投资关键作用，积极培育新的经济增长点

新常态下，投资仍将是拉动我市经济增长的主要动力。在传统投资领域相对饱和的情况下，迫切需要寻找新的投资增长点。要发挥好传统投资渠道作用，不断创新投融资方式，有效释放社会投资潜力，民间投资占全部投资比重达到60%以上。借助国家加大对中西部和少数民族地区扶持力度的机遇，积极争取重点产业、基础设施、社会事业等领域的项目投资，上级投资额度要高于全区平均水平。创新招商引资方式，推动以商招商、定向招商，形成政府投资、社会投资、招商引资的聚合效应。全年实施亿元以上项目341个，当年完成投资800亿元。重点产业方面，实施农牧业项目30个，完成投资48亿元；工业项目113个，完成投资344亿元；商贸物流项目39个，完成投资86亿元。基础设施方面，实施重点项目119个，完成投资228亿元。交通建设重点推进科卓、呼凉丰一级公路等17个项目，集宁绕城高速全线通车。农村牧区公路建设和街巷硬化分别完成2000公里，嘎查村通沥青水泥路率达到85%以上，街巷硬化三年任务两年完成。推进城乡公交一体化，嘎查村通班车率达到100%。配合好呼张客专建设，集宁高铁车站全面开工。支线机场正式通航，凉城通用机场开工建设。电力、水利重点实施集宁西等17个电网和输变电工程，新增容量165万千伏安，七家营等3座中型水库基本完工。社会民生方面，实施重点项目40个，突出抓好扶贫攻坚、“十个全覆盖”等重大工程，建设基础教育、基层卫生、公共文化服务和保障性住房等重点项目，完成投资94亿元。

（三）大力发展现代农牧业，促进农牧民稳定增收

坚持收缩转移、集约发展，打好绿色牌、走好特色路，实现农牧业增产、农牧民增收。

提高规模化发展水平。以建设国家农业科技园区为抓手，加快奶牛、肉羊、蔬菜等6个示范园建设，形成“一区多园、覆盖全市”的经营构架。建设全国重要的马铃薯种薯供应基地，实施种薯质量全程监控，创建全国马铃薯种薯知名品牌示范区，新增种薯20万亩，达到120万亩。以改善结构、提升品质为重点，推广优质专用薯80万亩，建设马铃薯标准化生产基地100万亩。建设面向首都的冷凉蔬菜供应基地，重点打造前旗和商都两个50平方公里的冷凉蔬菜产业园，提升红萝卜、西芹等特色园区建设水平，建设露地蔬菜标准化生产基地3万亩。冷凉蔬菜达到80万亩，其中设施蔬菜19万亩。建设绿色畜禽产品供应基地，建成“百千万”规模化养殖园区（场）100处，创建畜禽标准化养殖场30处。启动杜蒙肉羊新品种培育工程，重点打造四子王旗赛诺、察右后旗蒙多利两个百万只肉羊养殖联合体，杜蒙杂交羔羊年生产量达到30万只。扶持奶牛产业发展，新增奶牛3万头，规模化养殖比例达到80%。推行肉牛杂交改良技术，年出栏肉牛20万头。引进扶持生猪肉鸡龙头企业，雏鹰集团300万口生猪产业化项目开工建设，四子王旗獭兔产业园项目达产达效。养殖业占农牧业增加值比重达到61%。

提高产业化经营水平。发展新型经营主体，培育专业种养大户，建设家庭农牧场示范点50个。出台奖励扶持政策，创建自治区级专业合作示范社30个，推进“三位一体示范社”建设。健全县乡村土地草牧场流转服务平台，流转面积分别增加80万亩、100万亩。创新利益连接机制，以产业集群发展为方向，推行“股份合作”、“订单合同”等经营模式，构建龙头企业与农牧民“风险共担、利益共享”共同体。整合提升龙头企业，双汇集团15万吨火腿肠加工项目开工建设，民丰薯业万吨马铃薯全粉项目投产达效，集宁双汇、商都蓝威斯顿、民丰薯业成为自治区领军企业。农畜产品加工转化率达到52%。

提高品牌化建设水平。制定薯、菜、乳、肉主导品种生产技术标准，强化金融、科技、气象、农机等方面支撑，提高标准化生产水平。建立农畜产品质量安全检验监测全覆盖体系和可追溯体系，将民丰等10家企业纳入自治区可追溯试点。认证无公害、绿色、有机农畜产品30个，新增认证面积10万亩。“四子王肉羊”、“商都西芹”申报国家地理标志证明商标，“鹏亚皮件”申报中国驰名商标。

（四）做大做强工业经济，提高发展质量和效益

坚持存量提质、增量做优，推动传统产业新型化、新兴产业规模化、支柱产业多元化，打造工业经济升级版。工业固定资产投资和规模以上工业增加值分别增长13%和11%。

优先发展能源产业。坚持以火电引领风电、光电，新增电力装机230万千瓦，把电力打造成全市最重要的支柱产业，建设保障首都、服务华北的重要电力输出基地。火电新增装机140万千瓦，集宁、兴和2个热电项目建成投产，卓资、兴和、后旗等火电项目开工建设。风电新增装机70万千瓦，支持火电和风机制造企业发展风电项目，三峡、内能建等项目加快建设。光伏发电新增装机20万千瓦，支持光农结合、分布式光伏项目发展，后旗奥特维斯等项目投入运行。天然气形成日处理540万立方米液化能力，抓好陕京四线呼兴天然气管道建设，加快卓资、兴和5个天然气项目建设。

改造提升传统产业。坚持装备现代化、产业高端化、生产循环化，着力推进产业转型升级。氟化工要进一步扩大四子王永和氟化工生产规模，推进丰镇氟化工园区发展循环经济，扩大聚偏氟乙烯、新型制冷剂等高端产品生产能力，开发高性能氟橡胶等系列产品，实现由初中级产品向高终端产品转变，形成30万吨的综合生产能力。石墨化工要尽快实现瑞盛石墨二期工程投产，重点发展石墨烯、石墨纸、金刚石等高端系列产品，力争走在行业前列，综合产能达到30万吨以上。电石化工要扩大PVC、PVA、糊状树脂和特种纤维以及高档型材系列产品生产规模，推进产业由原料初加工型向高端产品生产型转变，形成450万吨以上的综合产能。冶金产业要重点推动丰镇上泰特种合金项目投产达效，化德天成10万吨硅锰合金和余热利用项目开工建设，向标准件铸造、精密合金冶炼和型材加工等终端产品延伸。坚持“上大压小、等量或减量置换”，淘汰1.25万千伏安及以下矿热炉。四子王旗和凉城县不再新上高载能项目。建成全市工业能耗在线监测平台，年耗能3000吨标煤以上企业全部纳入管理。建材产业要提高石材行业准入门槛，重点建设四子王、中旗、化德采选加一体化示范园区。推动森诺木材加工园区一期项目投产达效，打造木材初级产品加工和家具材料生产基地。大力发展以工业废渣为原料的新型墙体材料。装备制造业要鼓励风电企业优先使用地产风机，推动太重等风机制造项目建成投产，整机总装能力达到1850台（套）。

培育壮大云计算产业。抓住北京地区不再审批大数据中心、华为公司把我市作为云计算中心一级节点的有利机遇，编制云计算产业发展规划，大力发展电子商务、物联网、政务服务等云产业，建设“智慧乌兰察布”，打造华北地区重要的大数据中心，把信息产业培育成全市的支柱产业。

提高园区建设管理水平。设立1000万元扶持资金，以奖代补，引导项目集中、产业集聚。坚持“一园一主业、园区有特色”，鼓励园区做大做强主导产业和龙头企业，培育石墨碳素、羊驼绒服装等特色产业园。8个旗县级园区升级为市级园区，争取1—2个市级园区升级为自治区级园区。旗下营工业园区打造自治区级循环经济产业园区，销售收入超百亿元。

（五）壮大现代服务业，推动产业结构优化升级。

把服务业作为转型升级的突破口，加大扶持培育力度，第三产业增加值占GDP比重达到35%。

继续壮大现代物流业。围绕打造区域性商贸物流中心，大力实施“四港一体”战略，发展物流配送、公铁航联运、第三方和第四方物流，加快保税仓库、保税物流园建设，构建辐射周边、大进大出的物流产业格局。“三个交易中心”实现营业收入85亿元。煤炭交易中心。乌兰察布综合物流产业园“三线一站一场”建成运营，推进煤炭生产运输清洁化、标准化示范区项目建设，国家级煤炭质检中心建成运营。西北煤炭电子交易平台全面上线。引进张家口等地煤炭经销企业，打造北方最大的煤炭交易市场。煤炭吞吐量达到1000万吨，营业收入50亿元。马铃薯交易中心。实施民丰薯业、嘉恒农科等大型标准化冷藏项目，建设京津地区农产品销地集配中心。马铃薯年交易量达到200万吨，营业收入20亿元。皮革交易中心。编制完成全市皮革产业发展规划，土牧尔台制革基地和鑫源通皮件加工孵化园全面启动。皮革营业收入突破15亿元。

积极发展商贸流通业。深入实施“新网工程”。新增便民连锁超市249家，规范配送中心运营管理。完成中心城区商业网点规划，恩和世纪广场等商贸综合体投入使用。启动全市电子商务规划编制工作，重点建设煤炭、马铃薯、红萝卜等电子信息平台，每个旗县至少有一家电商进入。在农村牧区建设21个邮政综合服务平台试点，为农牧民提供邮政公共服务和快递物流服务。

加快发展文化旅游业。围绕打响“中国草原避暑之都”品牌，推动旅游产业提质升级，打造京津冀和呼包鄂知名旅游目的地。抓好重点景区建设。完善中心城区旅游集散功能，京蒙游客服务中心建成运营。加快凉城旅游献礼工程建设，完善岱海湖、温泉小镇旅游配套设施，蛮汉山申报国家地质公园。提升辉腾锡勒景区经营管理水平。岱海、格根塔拉创建5A级景区。后旗火山草原景区开业运营。加快开发苏木山、红召等旅游资源。加强旅游促销和行业管理。抓住京张联合申奥的有利机遇，做好旅游宣传推介，加强与京津冀的深度合作。推动乌大张旅游联盟一体化和全市场营销，实现惠民旅游一卡通。开辟中俄蒙“草原丝路”旅游精品线路。强化旅游市场管理，规范旅游消费环境，提升旅游服务水平。推进文化旅游深度融合。深入挖掘具有地区特色的文化资源，精心打造集宁路遗址文化园、绥蒙革命纪念园等文化旅游项目，推出一批特色经典剧目，提升旅游品位和整体形象。全年接待游客1200万人次，旅游收入超百亿元。

大力培育新兴服务业。准确把握新常态下消费需求的新变化，积极培育“四大新兴产业”，打造扩大投资的新领域、增加就业的新空间，形成新的经济增长极。发展文化休闲产业，依托得天独厚的自然环境和人文资源，推出一批独具民族地域特色的文化产业和文艺作品，打造创作演出基地，使“民族风”成为乌兰察布又一文化名片。促进养老养生产业，制定养老服务业发展意见，推进医养一体化，打造“夕阳红”养老服务品牌。繁荣体育健身产业，加快发展民族传统体育、体育健身等产业，打造体育强市。拓展医疗保健产业，推行职高与医院联办专业、联合培训，打造高护、特护、康复品牌。（六）加快新型城镇化进程，促进城乡一体化发展。

顺应京津冀城市群规划布局，承接首都城市功能疏解，打造吸引人口、吸纳资源、吸附产业的城市带和经济走廊。

优化城镇布局和形态。围绕自治区城镇体系规划和长城金三角合作区建设，编制全市城乡一体化城镇体系规划，推动国民经济和社会发展规划、城市规划和土地利用总体规划“多规合一”，促进产城一体化和区域合作互动，构筑层级合理、多点支撑、良性互动的城镇发展格局。中心城区按照“一市两区三组团”发展架构，做好城市出入口、沿霸王河及交通干线等重点地段节点的规划设计工作。旗县市以扩容提质为重点，完善控制性详规、专项规划和城市设计。严格实施城乡规划，保持规划的权威性、连续性、协调性。

提升城镇发展水平。以“五城联创”为抓手，加快沿路沿线城镇和园区建设，打造生产集约高效、生活宜居适度、生态山清水秀的现代城市。完善城镇功能。老城区通过棚户区和城中村改造，减少危旧房和城市污染，腾出空间增加公园绿地和公共服务设施，改善人居环境。新城区重点加强市政基础设施建设，统筹配套文化、教育、医疗、商业等公共设施，推进工业、物流等产业园区建设，提高人口吸纳和产业承载能力。全市实施市政重点项目282项，完成投资52亿元。建设城镇道路119公里。启动城镇地下市政管网普查。加强热源厂和供热管网建设，集中供热普及率达到87%。加快实施天然气进城入户工程。中心城区新开通公交线路2条，新增公交车50台，出租车全部实现公司化运营。建设马青地等一批水源地，新建改造供水管网209公里。提升城镇品位。发展绿色建筑和住宅全装修，可再生能源规模化示范城市通过国家验收，争取列入国家住宅产业现代化试点城市。深入开展建设工程质量治理两年行动，规范建筑市场管理。中心城区重点抓好机场路及新区14条道路的景观绿化工程，争创国家园林城市。凉城等7个旗县市争创自治区园林县城。大力推进数字城管建设，抓好物业管理升级达标工作，提高精细化管理水平。

建设美丽村庄。认真落实“十个全覆盖”工程三年规划，坚持实际实用的原则，全市70%的嘎查村达到“十个全覆盖”，所有嘎查村（互助幸福院）实现互联互通惠农便民金融服务全覆盖。注重典型引路，提升管理和服务水平，因地制宜建设一批样板工程。

（七）推进生态文明建设，构筑北疆生态安全屏障

加强生态建设与保护。抓好京津风沙源等国家重点生态建设工程，推进园区、城镇、“十个全覆盖”嘎查村等重点区域绿化工程。完成林业生态建设100万亩、水保治理面积105万亩，草原建设总规模达到500万亩。落实草原生态补奖机制，严格实施禁牧和草畜平衡制度。实行“一把手”负责制、责任追究制和限期整改制，加大矿山、河道、园区地质环境恢复治理力度。加强生态文明制度建设，开展四子王旗“国家主体功能区”建设试点示范工程。

加大节能减排力度。严格执行新《环保法》，严厉查处各类环境违法行为。抓好大气污染防治，持续改善空气质量。加强污水管网建设，生活污水处理厂负荷率达到60%以上。实施污水处理厂中水回用工程，提高水资源利用效率。实施用能预算管理，强化节能减排责任考核，全面完成“十二五”节能减排目标。

集约节约利用资源。实行最严格的水资源管理和耕地保护制度。加大废弃居民点和工矿废弃地复垦项目实施力度，促进国土资源节约和高效利用。加大地勘工作力度，加快地勘项目成果转化应用。

（八）着力保障和改善民生，提高人民群众生活水平。

把民之所望作为施政所向，优先保障民生投入，优先安排民生项目，优先解决民生问题，让发展成果更多更公平地惠及全市人民。

加大扶贫开发力度。把扶贫开发作为头号民生工程，统筹推进“三三制”扶贫战略。实行“三到村三到户”精准扶贫，完成145个重点贫困嘎查村的整村推进。发挥扶贫资金的杠杆和引导作用，发放金融扶贫富民贷款17亿元、农户小额贷款2亿元。以整村推进项目资金为风险担保金，在部分嘎查村进行生产性贷款试点。完成生态移民扶贫搬迁1万人。继续实施“暖心煤”工程。扶贫现金直补标准提高到2000元。对当年入学的3000名贫困家庭高职、中职、中专学生每人一次性补贴1500元。扎实开展“扶贫日”活动，构建多元化社会扶贫格局，实现10万人稳定脱贫，人均纯收入增长20%。

推进就业创业工程。实施更加积极的就业政策，健全公共就业服务体系，做好高校毕业生等重点群体就业工作。鼓励支持自主创业，发挥创业带动就业倍增效应，发放小额担保贷款1.5亿元，带动就业6000多人。

实施“金蓝领”培训计划，打造更多劳务品牌，免费培训城乡劳动者1万名，新建市级标准化创业园4个、市级就业实训基地6个。转移农牧民就业30万人，新增城镇就业1.7万人，城镇登记失业率控制在4%以内。

完善社会保障体系。全面推行农村牧区社会保障信息化服务互联互通，提高便民惠民整体水平。完善城乡居民养老保险制度，做好企业退休人员基本养老保险待遇调整工作。提高城乡医疗保障水平。启动城镇医疗保险异地就医即时结算试点工作。推广察右前旗门诊统筹补偿模式，提高参合农牧民报销水平。城镇职工和居民门诊慢性病报销病种增加到30种。参合农牧民门诊慢性病报销病种增加到35种，慢性病和特种病门诊报销封顶线由1万元提高到1.2万元。健全社会救助体系，提高城乡低保和五保供养水平。给予困境儿童和困境家庭儿童每人每月505元救助。

推进百姓安居工程。探索回购商品房用于保障性住房等模式，推进公共租赁住房和廉租住房制度并轨运行，新开工保障性住房2.5万套，基本建成2.8万套。统筹推进农村牧区危房改造和村庄整治，完成3.9万户危房改造任务。加强互助幸福院配套设施建设，提高管理服务水平。

加强社会管理创新。完善社会治安防控体系，加快视频监控“天网”工程建设，严密防范、严厉打击各类违法犯罪活动。开展“基层组织带头人队伍建设年”活动，提升城乡社区治理和服务水平。加强安全生产基层基础建设，建立安全生产长效机制。巩固完善食品药品安全监管体系，建设“食品药品安全乌兰察布”。中心城区实现食品生产、流通和餐饮示范创建全覆盖，县城实现餐饮示范创建全覆盖。实施名牌战略，启动“市长质量奖”。依法做好第九届嘎查村（居）民委员会换届选举工作。完善应急管理机制。实行重大事项社会稳定风险评估制度，有效预防和化解社会矛盾。抓好国防教育和双拥工作，巩固发展军政军民团结。加强人防、地震等防灾减灾工作。

（九）统筹发展社会事业，推进基本公共服务均等化

优先发展教育事业。继续深化中小学和幼儿教育“三项改革”，启动第二轮校长聘任和教师全员竞聘上岗，开展“全市课改年”活动，提升教育改革活力。实施“全面改薄”项目，加快义务教育学校标准化建设，启动4个旗县义务教育均衡发展水平评估。推进普通高中优质、多样化发展，提升办学水平和质量，北京八中分校通过自治区示范学校验收。农村牧区公办幼儿园全部实行免费入园，新建公办幼儿园12所，学前三年入正规园率提高到75%。积极调整职业教育办学模式，优化区域专业布局，整合资源、形成优势。优先发展民族教育，鼓励扶持民办教育，关心支持特殊教育，加快高等教育内涵发展。

大力发展医疗卫生事业。整合卫生、计生资源，加快苏木乡镇、嘎查村社区卫生院建设，推进医疗卫生服务网络全覆盖，为群众就医提供便利服务。市第二医院、妇幼保健院和地病中心投入使用。每个旗县市建成1所政府主导的标准化社区卫生服务中心。全面推进县级公立医院综合改革。开展“医疗质量提升年”活动，加强医疗机构内涵建设，凉城县医院等5家医院创二甲，市中心医院创三甲。深化京蒙医疗帮扶合作，实现每个旗县一家医院与京沪等地医疗机构建立技术协作关系。

全面发展科技文体等事业。加快科技创新，加强“两院一站”建设，瑞盛天然石墨和朋诚良种猪繁育两个院士工作站启动运行，推动科技与产业深度融合。深入实施文化惠民工程，组织开展丰富多彩的群众文化活动，推进文化场所便利化、网格化，打造“15分钟文化生活休闲圈”。更加注重对地区特色文化的研究和开发，更加注重察哈尔婚礼等非物质文化遗产的传承和保护，创作完成自治区成立70周年献礼剧目。整合现有资源，统筹推进体育公园、场馆和设施改造建设，形成举办综合赛事的能力。做好十四运筹备工作。以建设足球训练基地为突破口，与自治区、京津冀和国家相关部门及团体加强对接，建设一批国家级和区域性体育训练基地。落实好计划生育政策。深入开展民族团结进步创建活动。统筹发展妇女儿童、残疾人等事业，促进社会全面进步。

各位代表，党的十八届三中、四中全会吹响了全面深化改革、加强法治建设的号角，对政府自身建设提出了更高要求，我们将准确把握改革发展新要求和人民群众新期待，解放思想、勇于担当，努力提高政府治理能力。一要坚持依法行政，着力建设法治政府。坚持法无授权不可为、法定职责必须为，自觉运用法治思维、法治方式和法治手段履行政府职能。健全重大行政决策机制，严格行政执法程序，实行行政执法人员持证上岗和资格管理制度，加快建设职能科学、权责法定、执法严明、公开公正、守法诚信的法治政府。二要坚持勤政为民，着力建设服务政府。巩固教育实践活动成果，强化宗旨意识、服务意识，深入基层，问政于民、问需于民、问计于民，多谋民生之利，多解民生之忧，最大限度地把群众利益实现好、保护好、发展好。三要坚持求真务实，着力建设实干政府。发扬苦干实干精神，把更多精力用在谋划发展、推动工作上。加强对重大决策、重点项目的督查落实，提高政府执行力，营造想干事、会干事、干成事的浓厚氛围。四要坚持廉洁从政，着力建设廉洁政府。深入推进政府系统惩防体系建设，认真落实党风廉政建设主体责任，把廉政建设要求落实到政府工作各个环节。加大行政监察和审计监督力度，强化行政问责。全面压缩“三公经费”，完成公务用车改革，把更多财力用于保障和改善民生，政府自觉过“紧日子”，让群众过上“好日子”。

在抓好今年各项工作的同时，着眼于经济发展新常态，科学编制“十三五”规划，引领“十三五”持续健康发展。

各位代表，事业凝聚人心，实干赢得未来。全市人民的信任与支持，是我们勇于担当、奋勇向前的信心所在和动力源泉。让我们在市委的坚强领导下，紧紧团结和依靠全市各族人民，以更加务实的作风、更加昂扬的斗志、更加饱满的热情，解放思想、开拓创新，守望相助、团结奋斗，为全面实现“十二五”规划目标，建设祖国北疆亮丽风景线而努力奋斗！
